# Crystal structure of di-μ-iodido-bis{[bis(piperidin-1-yl)methane-κ^2^ *N*,*N*′]copper(I)}

**DOI:** 10.1107/S2056989015018757

**Published:** 2015-10-14

**Authors:** Eva Rebecca Barth, Christopher Golz, Carsten Strohmann

**Affiliations:** a Fakultät für Chemie und Chemische Biologie, Technische Universität Dortmund, Otto-Hahn-Strasse 6, 44227 Dortmund, Germany

**Keywords:** crystal structure, copper iodide, dimer, small ring, four-membered chelate ring.

## Abstract

The title compound, [Cu_2_I_2_(C_11_H_22_N_2_)_2_], crystallizes as a symmetric dimer with one quarter of the mol­ecule in the asymmetric unit. The copper(I) atom, the iodine atom and the central methyl­ene C atom of the di(piperidin-1-yl)methane ligand lie on a mirror plane and the complete molecule exhibits point group symmetry 2/*m*. To the best of our knowledge it is the first di­amine copper(I) complex containing a four-membered chelate ring. Compared to other di­amine copper(I) iodide dimers, the title compound has a short Cu⋯Cu distance of 2.5137 (11) Å, but a long Cu—N bond length of 2.213 (3) Å. The I—Cu—I angle [121.84 (2)°] is large, and the N—Cu—N angle = 66.61 (13)° is the smallest one found for copper(I) di­amine complexes. As a result of the four-membered ring, the ligands around the copper(I) atom have an extremely distorted tetra­hedral arrangement. In the crystal, there are no significant inter­molecular inter­actions present.

## Related literature   

To the best of our knowledge no related di­amine complexes with four-membered chelate rings are known. For di­amine complexes with five-membered chelate rings, see: Haitko (1984 ▸); Garbauskas *et al.* (1986 ▸). For a bi­pyridine complex containing a copper(I) iodide dimer, see: Huang *et al.* (2013 ▸). For the crystal structure of the μ,μ′-diiodido-bridged dimer, with four-coordinate copper(I), *viz.* [(py)_2_CuI_2_Cu(py)_2_] (py is pyridine), see: Dyason *et al.* (1984 ▸).
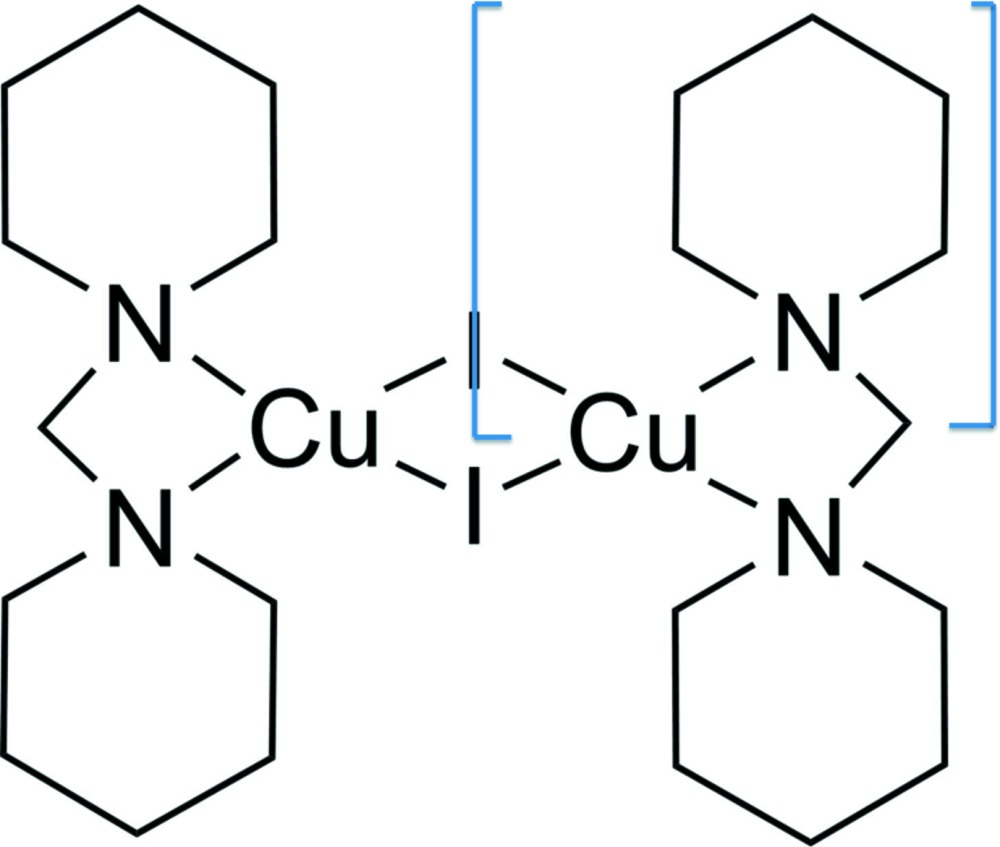


## Experimental   

### Crystal data  

[Cu_2_I_2_(C_11_H_22_N_2_)_2_]
*M* *_r_* = 745.49
Orthorhombic, 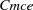

*a* = 18.718 (4) Å
*b* = 8.4175 (15) Å
*c* = 17.074 (3) Å
*V* = 2690.1 (8) Å^3^
*Z* = 4
Mo *K*α radiation
μ = 3.89 mm^−1^
*T* = 173 K
0.4 × 0.3 × 0.2 mm

### Data collection  

Bruker APEXII CCD diffractometer
Absorption correction: multi-scan (*SADABS*; Krause *et al.*, 2015 ▸) *T*_min_ = 0.256, *T*_max_ = 0.459
38065 measured reflections
1704 independent reflections
1349 reflections with *I* > 2σ(*I*)
*R*_int_ = 0.096

### Refinement  

*R*[*F* ^2^ > 2σ(*F* ^2^)] = 0.032
*wR*(*F* ^2^) = 0.070
*S* = 1.07
1704 reflections
73 parameters
H-atom parameters constrained
Δρ_max_ = 0.99 e Å^−3^
Δρ_min_ = −0.44 e Å^−3^

### 

Data collection: *APEX2* (Bruker, 2003 ▸); cell refinement: *SAINT* (Bruker, 2003 ▸); data reduction: *SAINT*; program(s) used to solve structure: *SHELXT* (Sheldrick, 2015*a* ▸); program(s) used to refine structure: *SHELXL2014*/6 (Sheldrick, 2015*b* ▸); molecular graphics: *Mercury* (Macrae *et al.*, 2008 ▸); software used to prepare material for publication: *OLEX2* (Dolomanov *et al.*, 2009 ▸) and *publCIF* (Westrip, 2010 ▸).

## Supplementary Material

Crystal structure: contains datablock(s) Global, I. DOI: 10.1107/S2056989015018757/su5211sup1.cif

Structure factors: contains datablock(s) I. DOI: 10.1107/S2056989015018757/su5211Isup2.hkl

**Fig. 1.**
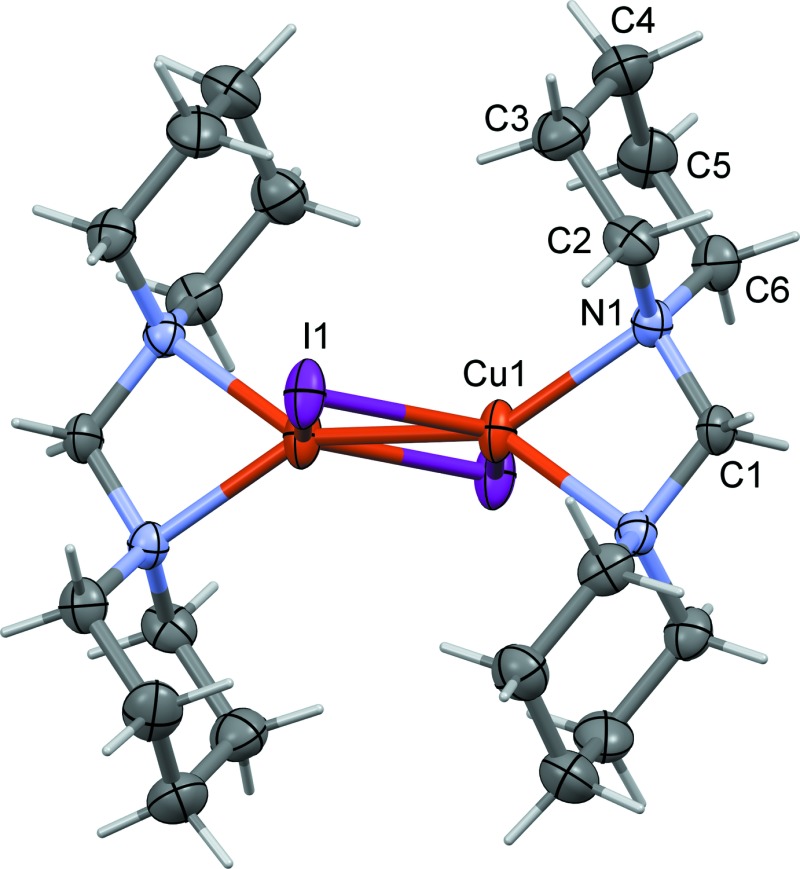
Molecular structure of the title compound, with atom labelling. The displacement ellipsoids are drawn at the 50% probability level.

**Fig. 2.**
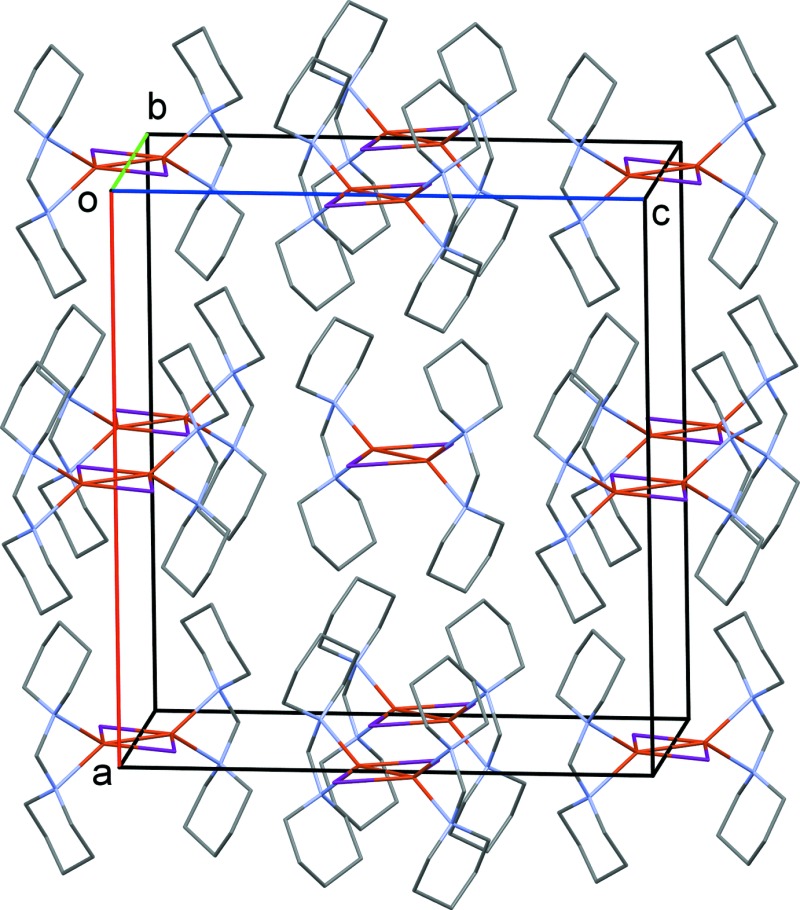
Crystal packing of the title compound viewed along b axis. H-atoms have been omitted for clarity.

. DOI: 10.1107/S2056989015018757/su5211fig1.tif
Mol­ecular structure of the title compound, with atom labelling. The displacement ellipsoids are drawn at the 50% probability level.

b . DOI: 10.1107/S2056989015018757/su5211fig2.tif
Crystal packing of the title compound viewed along *b* axis. H-atoms have been omitted for clarity.

CCDC reference: 1429684

Additional supporting information:  crystallographic information; 3D view; checkCIF report

## Figures and Tables

**Table:** 

Cu1Cu1^i^	2.5137(11)
I1Cu1	2.5798(8)
I1Cu1^i^	2.5922(7)
Cu1N1	2.213(3)

**Table:** 

Cu1I1Cu1^i^	58.16(2)
N1Cu1N1^ii^	66.61(13)
N1Cu1I1	116.02(7)
N1Cu1I1^i^	111.90(7)
I1Cu1I1^i^	121.84(2)

Symmetry codes: (i) 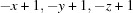; (ii) 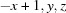.

## supplementary crystallographic information

### S1. Synthesis and crystallization

The title compound was prepared by dissolving 98 mg (0.51 mmol) of CuI in 2 ml acetone under an inert argon atmosphere. Then 151 mg (0.83 mmol) of di(piperidin-1-yl)methane was added to this solution with stirring. After one week colourless crystals of the title compound were obtained.

### S2. Refinement

Crystal data, data collection and structure refinement details are summarized in Table 2. The C-bound H atoms were included in calculated positions and treated as riding atoms: C—H = 0.99 Å with U_iso_(H) = 1.2U_eq_(C).

### Crystal data

**Table:**

[Cu_2_I_2_(C_11_H_22_N_2_)_2_]	*D*_x_ = 1.841 Mg m^−^^3^
*M**_r_* = 745.49	Mo *K*α radiation, λ = 0.71073 Å
Orthorhombic, *C**m**c**e*	Cell parameters from 38065 reflections
*a* = 18.718 (4) Å	θ = 2.2–28.2°
*b* = 8.4175 (15) Å	µ = 3.89 mm^−^^1^
*c* = 17.074 (3) Å	*T* = 173 K
*V* = 2690.1 (8) Å^3^	Block, colourless
*Z* = 4	0.4 × 0.3 × 0.2 mm
*F*(000) = 1472	

### Data collection

**Table:**

Bruker APEXII CCD diffractometer	1349 reflections with *I* > 2σ(*I*)
φ and ω scans	*R*_int_ = 0.096
Absorption correction: multi-scan (*SADABS*; Krause *et al.*, 2015)	θ_max_ = 28.2°, θ_min_ = 2.2°
*T*_min_ = 0.256, *T*_max_ = 0.459	*h* = −24→24
38065 measured reflections	*k* = −11→11
1704 independent reflections	*l* = −22→22

### Refinement

**Table:**

Refinement on *F*^2^	0 restraints
Least-squares matrix: full	Hydrogen site location: inferred from neighbouring sites
*R*[*F*^2^ > 2σ(*F*^2^)] = 0.032	H-atom parameters constrained
*wR*(*F*^2^) = 0.070	*w* = 1/[σ^2^(*F*_o_^2^) + (0.0354*P*)^2^ + 0.6685*P*] where *P* = (*F*_o_^2^ + 2*F*_c_^2^)/3
*S* = 1.07	(Δ/σ)_max_ = 0.001
1704 reflections	Δρ_max_ = 0.99 e Å^−^^3^
73 parameters	Δρ_min_ = −0.43 e Å^−^^3^

### Special details

**Table:**

Geometry. All esds (except the esd in the dihedral angle between two l.s. planes) are estimated using the full covariance matrix. The cell esds are taken into account individually in the estimation of esds in distances, angles and torsion angles; correlations between esds in cell parameters are only used when they are defined by crystal symmetry. An approximate (isotropic) treatment of cell esds is used for estimating esds involving l.s. planes.

### Fractional atomic coordinates and isotropic or equivalent isotropic displacement parameters (Å^2^)

**Table:**

	*x*	*y*	*z*	*U*_iso_*/*U*_eq_	
I1	0.5000	0.70816 (3)	0.58360 (2)	0.03716 (12)	
Cu1	0.5000	0.40635 (6)	0.55733 (3)	0.03283 (16)	
N1	0.56490 (13)	0.2603 (3)	0.63824 (16)	0.0259 (5)	
C1	0.5000	0.1792 (5)	0.6679 (3)	0.0299 (10)	
H1A	0.5000	0.0670	0.6505	0.036*	
H1B	0.5000	0.1806	0.7259	0.036*	
C2	0.60139 (18)	0.3498 (4)	0.70093 (19)	0.0333 (7)	
H2A	0.6165	0.2756	0.7427	0.040*	
H2B	0.5677	0.4274	0.7241	0.040*	
C3	0.66608 (19)	0.4367 (4)	0.6694 (2)	0.0444 (9)	
H3A	0.6504	0.5176	0.6310	0.053*	
H3B	0.6905	0.4924	0.7129	0.053*	
C4	0.7177 (2)	0.3238 (5)	0.6306 (3)	0.0497 (10)	
H4A	0.7382	0.2510	0.6702	0.060*	
H4B	0.7573	0.3844	0.6064	0.060*	
C5	0.6785 (2)	0.2285 (5)	0.5680 (2)	0.0460 (9)	
H5A	0.6628	0.3006	0.5255	0.055*	
H5B	0.7114	0.1490	0.5451	0.055*	
C6	0.61428 (18)	0.1448 (4)	0.60214 (19)	0.0333 (8)	
H6A	0.5891	0.0858	0.5603	0.040*	
H6B	0.6302	0.0674	0.6421	0.040*	

### Atomic displacement parameters (Å^2^)

**Table:**

	*U*^11^	*U*^22^	*U*^33^	*U*^12^	*U*^13^	*U*^23^
I1	0.0678 (3)	0.02108 (15)	0.02262 (17)	0.000	0.000	−0.00298 (11)
Cu1	0.0540 (4)	0.0219 (3)	0.0225 (3)	0.000	0.000	0.0030 (2)
N1	0.0293 (13)	0.0222 (11)	0.0262 (13)	0.0010 (10)	0.0021 (11)	−0.0001 (10)
C1	0.032 (3)	0.028 (2)	0.029 (3)	0.000	0.000	0.0092 (18)
C2	0.0359 (19)	0.0369 (17)	0.0271 (17)	0.0032 (14)	−0.0048 (14)	−0.0078 (14)
C3	0.040 (2)	0.041 (2)	0.052 (2)	−0.0066 (16)	−0.0035 (17)	−0.0046 (17)
C4	0.036 (2)	0.051 (2)	0.062 (3)	−0.0062 (17)	0.0047 (19)	0.0075 (19)
C5	0.043 (2)	0.047 (2)	0.048 (2)	0.0054 (18)	0.0177 (17)	0.0000 (18)
C6	0.040 (2)	0.0289 (16)	0.0314 (18)	0.0033 (14)	0.0023 (15)	−0.0046 (13)

### Geometric parameters (Å, º)

**Table:**

Cu1—Cu1^i^	2.5137 (11)	C2—H2A	0.9900
I1—Cu1	2.5798 (8)	C2—H2B	0.9900
I1—Cu1^i^	2.5922 (7)	C3—C4	1.509 (5)
Cu1—N1	2.213 (3)	C3—H3A	0.9900
Cu1—N1^ii^	2.213 (3)	C3—H3B	0.9900
Cu1—I1^i^	2.5922 (7)	C4—C5	1.526 (6)
N1—C6	1.476 (4)	C4—H4A	0.9900
N1—C2	1.477 (4)	C4—H4B	0.9900
N1—C1	1.483 (3)	C5—C6	1.509 (5)
C1—N1^ii^	1.483 (3)	C5—H5A	0.9900
C1—H1A	0.9900	C5—H5B	0.9900
C1—H1B	0.9900	C6—H6A	0.9900
C2—C3	1.513 (5)	C6—H6B	0.9900
			
Cu1—I1—Cu1^i^	58.16 (2)	C3—C2—H2B	109.4
N1—Cu1—N1^ii^	66.61 (13)	H2A—C2—H2B	108.0
N1—Cu1—Cu1^i^	146.59 (7)	C4—C3—C2	111.4 (3)
N1^ii^—Cu1—Cu1^i^	146.59 (7)	C4—C3—H3A	109.4
N1—Cu1—I1	116.02 (7)	C2—C3—H3A	109.4
N1^ii^—Cu1—I1	116.01 (7)	C4—C3—H3B	109.4
Cu1^i^—Cu1—I1	61.17 (2)	C2—C3—H3B	109.4
N1—Cu1—I1^i^	111.90 (7)	H3A—C3—H3B	108.0
N1^ii^—Cu1—I1^i^	111.90 (7)	C3—C4—C5	109.3 (3)
Cu1^i^—Cu1—I1^i^	60.67 (3)	C3—C4—H4A	109.8
I1—Cu1—I1^i^	121.84 (2)	C5—C4—H4A	109.8
C6—N1—C2	110.4 (2)	C3—C4—H4B	109.8
C6—N1—C1	110.6 (3)	C5—C4—H4B	109.8
C2—N1—C1	111.5 (3)	H4A—C4—H4B	108.3
C6—N1—Cu1	116.7 (2)	C6—C5—C4	111.0 (3)
C2—N1—Cu1	115.02 (19)	C6—C5—H5A	109.4
C1—N1—Cu1	91.11 (18)	C4—C5—H5A	109.4
N1^ii^—C1—N1	110.0 (3)	C6—C5—H5B	109.4
N1^ii^—C1—H1A	109.7	C4—C5—H5B	109.4
N1—C1—H1A	109.7	H5A—C5—H5B	108.0
N1^ii^—C1—H1B	109.7	N1—C6—C5	110.6 (3)
N1—C1—H1B	109.7	N1—C6—H6A	109.5
H1A—C1—H1B	108.2	C5—C6—H6A	109.5
N1—C2—C3	111.0 (3)	N1—C6—H6B	109.5
N1—C2—H2A	109.4	C5—C6—H6B	109.5
C3—C2—H2A	109.4	H6A—C6—H6B	108.1
N1—C2—H2B	109.4		
			
C6—N1—C1—N1^ii^	−128.9 (3)	C2—C3—C4—C5	54.5 (4)
C2—N1—C1—N1^ii^	107.7 (3)	C3—C4—C5—C6	−55.1 (4)
Cu1—N1—C1—N1^ii^	−9.8 (3)	C2—N1—C6—C5	−59.2 (4)
C6—N1—C2—C3	58.5 (3)	C1—N1—C6—C5	176.9 (3)
C1—N1—C2—C3	−178.0 (3)	Cu1—N1—C6—C5	74.6 (3)
Cu1—N1—C2—C3	−76.1 (3)	C4—C5—C6—N1	57.9 (4)
N1—C2—C3—C4	−56.9 (4)		

Symmetry codes: (i) −*x*+1, −*y*+1, −*z*+1; (ii) −*x*+1, *y*, *z*.
