# Supplementary figures and images for: Crystal structure of di-μ-iodido-bis{[bis(piperidin-1-yl)methane-κ2 N,N′]copper(I)}
Source: Acta Crystallogr E Crystallogr Commun. 2015 Oct 14;71(Pt 11):m193–4. doi: 10.1107/S2056989015018757 (PMC4645043; doi:10.1107/S2056989015018757)

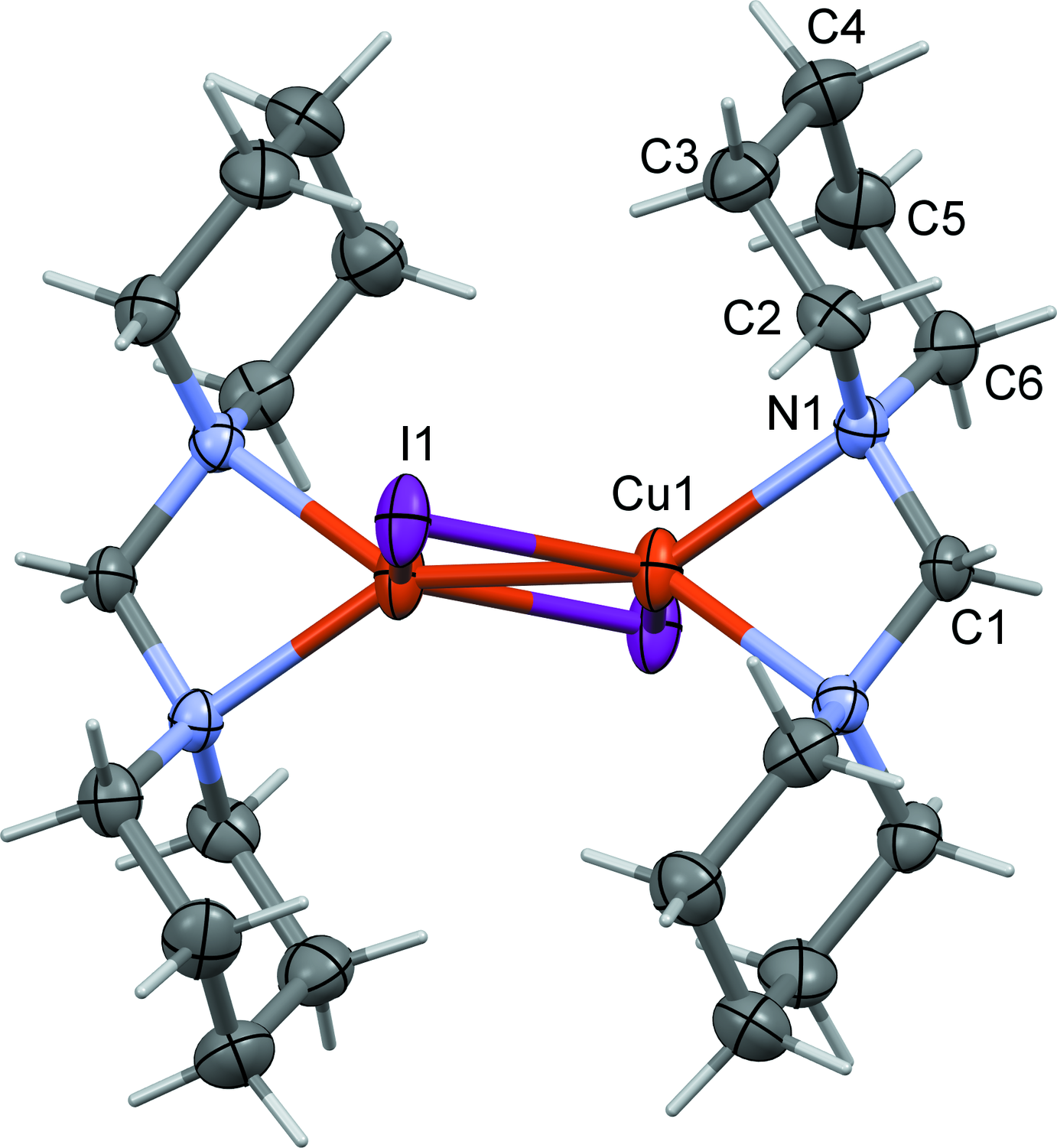

Supplement: Supplementary file 3 [file e-71-0m193-fig1.tif]

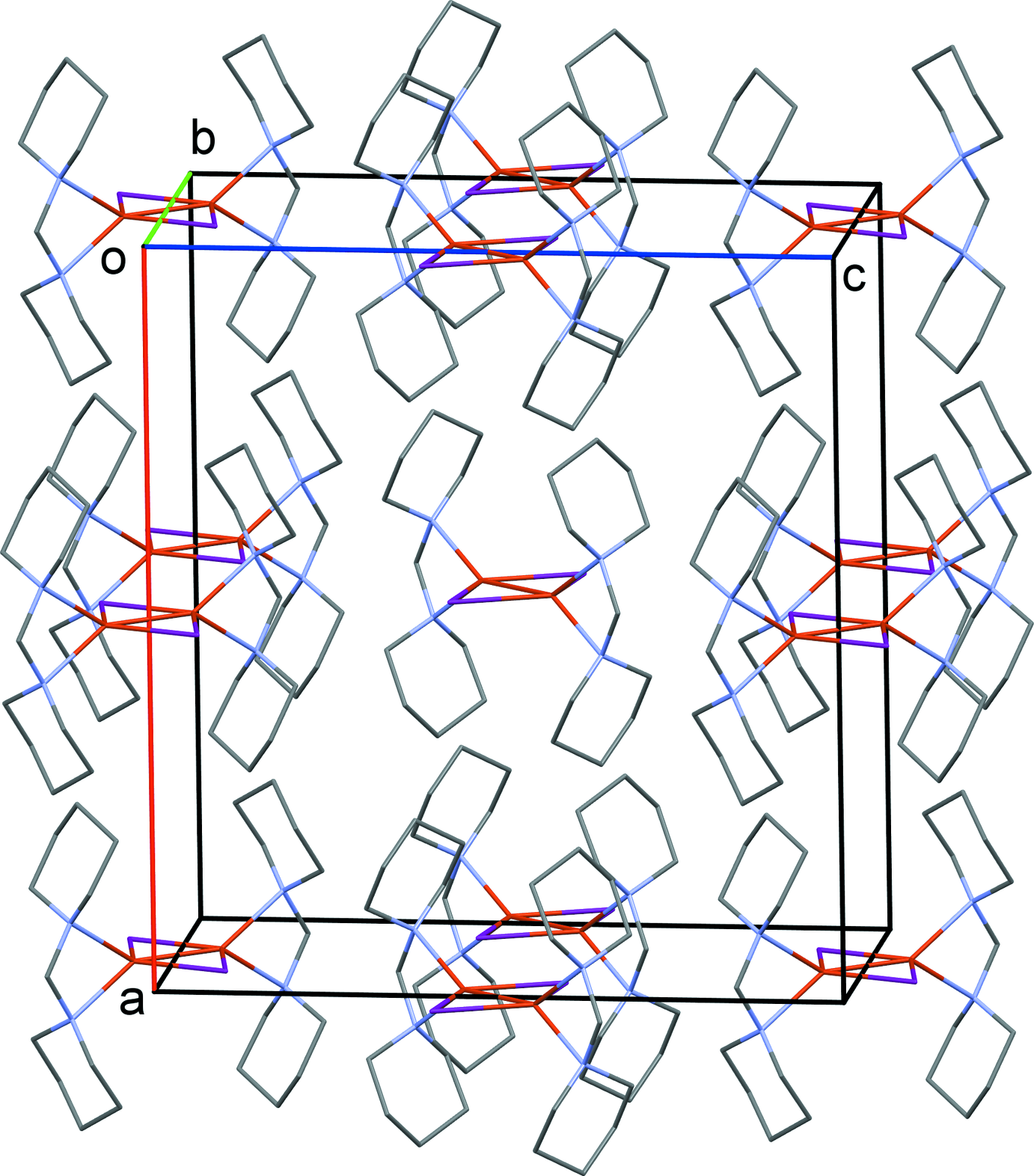

Supplement: Supplementary file 4 [file e-71-0m193-fig2.tif]
